# Reviewing the literature on access to prompt and effective malaria treatment in Kenya: implications for meeting the Abuja targets

**DOI:** 10.1186/1475-2875-8-243

**Published:** 2009-10-28

**Authors:** Jane Chuma, Timothy Abuya, Dorothy Memusi, Elizabeth Juma, Willis Akhwale, Janet Ntwiga, Andrew Nyandigisi, Gladys Tetteh, Rima Shretta, Abdinasir Amin

**Affiliations:** 1 Kenya Medical Research Institute/Wellcome Trust Programme P.O. Box 230, Kilifi, Kenya; 2 Division of Malaria Control, Ministry of Public Health and Sanitation, Kenya; 3 Management Sciences for Health, Strengthening Pharmaceutical Systems Programme, PO Box 8700-00100, Nairobi, Kenya

## Abstract

**Background:**

Effective case management is central to reducing malaria mortality and morbidity worldwide, but only a minority of those affected by malaria, have access to prompt effective treatment.

In Kenya, the Division of Malaria Control is committed to ensuring that 80 percent of childhood fevers are treated with effective anti-malarial medicines within 24 hours of fever onset, but this target is largely unmet. This review aimed to document evidence on access to effective malaria treatment in Kenya, identify factors that influence access, and make recommendations on how to improve prompt access to effective malaria treatment. Since treatment-seeking patterns for malaria are similar in many settings in sub-Saharan Africa, the findings presented in this review have important lessons for other malaria endemic countries.

**Methods:**

Internet searches were conducted in PUBMED (MEDLINE) and HINARI databases using specific search terms and strategies. Grey literature was obtained by soliciting reports from individual researchers working in the treatment-seeking field, from websites of major organizations involved in malaria control and from international reports.

**Results:**

The review indicated that malaria treatment-seeking occurs mostly in the informal sector; that most fevers are treated, but treatment is often ineffective. Irrational drug use was identified as a problem in most studies, but determinants of this behaviour were not documented. Availability of non-recommended medicines over-the-counter and the presence of substandard anti-malarials in the market are well documented. Demand side determinants of access include perception of illness causes, severity and timing of treatment, perceptions of treatment efficacy, simplicity of regimens and ability to pay. Supply side determinants include distance to health facilities, availability of medicines, prescribing and dispensing practices and quality of medicines. Policy level factors are around the complexity and unclear messages regarding drug policy changes.

**Conclusion:**

Kenya, like many other African countries, is still far from achieving the Abuja targets. The government, with support from donors, should invest adequately in mechanisms that promote access to effective treatment. Such approaches should focus on factors influencing multiple dimensions of access and will require the cooperation of all stakeholders working in malaria control.

## Background

Effective case management is central to reducing malaria mortality and morbidity worldwide. In the Abuja declaration of 2000, African heads of state committed themselves to ensure that 60 percent of childhood fevers are treated with effective anti-malarial medicines within 24 hours of symptom onset, a target that has since been increased to 80 percent by 2010 [1]. In Kenya, the Ministry of Health, together with the Division of Malaria Control (DOMC) are committed to achieving the Abuja targets, embedded within the strategic approaches of the National Malaria Strategy [2]. Together with partners, the DOMC have been monitoring progress toward these targets using both sentinel sites and national surveys.

Kenya has a multi-pronged malaria control strategy, through which the country has introduced artemisinin-based combination therapy (ACT) as the first-line drug [2,3], and dramatically increased the use of insecticide-treated nets among children under five [4]. Detailed guidelines for malaria case management were developed following the policy change from sulphadoxine-pyrimethamine (SP) to artemether-lumefantrine (AL). The World Health Organization (WHO) highlights the need to ensure access to and rational use of ACT, not only because ACT is more effective in case management, but also because irrational drug use could lead to drug resistance [5]. A summary of Kenya's treatment policy is provided in Table 1.

**Table 1:** Kenya's Current Malaria Treatment Policy

**Condition**	**Recommendation**	**Dosage Form**	**Strength**
Uncomplicated malaria (First-line treatment)	Artemether-lumefantrine	Tablet	20 mg Artemether + 120 mg Lumefantrine

Uncomplicated malaria (Second-line treatment)	Quinine	Tablet	200 mg 300 mg

Severe and complicated malaria			
Pre-referral treatment	Artemether injection	Injection	Adult: 80 mg/ml
			Pediatric: 20 mg/ml
	Artesunate injection	Injection	60 mg/1 ml ampoule
	Artesunate rectal caps	Suppositories	100 mg
			400 mg
			
IV/IM phase	Quinine	Injection (IV or IM)	300 mg/1 ml ampoule
			600 mg/2 ml ampoule
Continuation phase	Quinine	Tablet	200 mg
			300 mg

Prevention of malaria in pregnancy	Intermittent Preventive Treatment (IPTp) using SP	Tablet	Sulphadoxine 500 mg; Pyrimethamine 25 mg

Treatment of uncomplicated malaria in pregnancy	Trimester 1: Quinine	Tablet	300 mg
	Trimester 2 & 3: Quinine or	Tablet	300 mg
	Artemether-Lumefantrine	Tablet	20 mg Artemether + 120 mg Lumefantrine

Treatment of complicated malaria in pregnancy	The treatment of pregnant women with severe malaria shall be the same as the treatment of severe malaria in the general population.		

(Source: DOMC 2006)

Several initiatives have been implemented over the years to improve access to malaria treatment, including strengthening home management of fevers by training private medicine retailers on effective case management [6-10]; engaging community health workers to dispense anti-malarials [11-13]; and training health workers to diagnose and prescribe anti-malarials appropriately [14,15]. Some of these interventions have been shown to be effective [16], but their sustainability remains unknown. In an attempt to improve access to malaria treatment, the Kenyan government changed the charging policy at all public dispensaries and health centres in July 2004. User fees were abolished under the new policy and a flat registration fees introduced of Kenya Shillings (KES) 10 and KES 20 respectively (approximately 2008 USD 0.2 and 0.3 respectively). Children aged below five years and specific health conditions such as malaria were exempted from paying the registration fees. An evaluation of this policy showed that these changes were not fully implemented, and individuals suffering from malaria continued to pay the registration fees [17].

Although data on access to malaria treatment in Kenya is scanty, the majority of ill individuals, especially the poor, do not have access to prompt effective treatment [18]. Information on the factors that hinder people from seeking treatment promptly is lacking. While several studies have focused on treatment-seeking behaviour, there has been no attempt to synthesize existing data, or to create a comprehensive overview of the current access situation in Kenya. This paper seeks to document the evidence on access to effective malaria treatment in Kenya, identify factors that influence access, and identify gaps that should be addressed to improve prompt access to effective malaria treatment. Although the paper presents evidence from Kenya, the lessons learnt are useful for other malaria endemic countries in sub-Saharan Africa.

### Defining access

Most studies on access to malaria treatment focus on the ability to acquire anti-malarials and utilization of health care services. Yet access to prompt effective treatment is broader than utilization of services, and or being able to acquire anti-malarials. In this review, access is presented as a multifaceted issue, influenced by many interrelated factors occurring at the demand, supply, and policy level. Access comprises four main dimensions namely [19-22]: (1) availability, referring to the geographical location of health care services in relation to the clients; (2) affordability, which includes the costs of seeking care and ability to cope; (3) acceptability, referring to the nature of service provision and how individuals and communities perceive them and; (4) adequacy, which includes the extent to which the organization of health care meets client's expectation. To understand access and to be able to draw concrete and actionable policy recommendations, all elements of access should be considered comprehensively.

## Methods

### Search strategy

**Figure 1:**
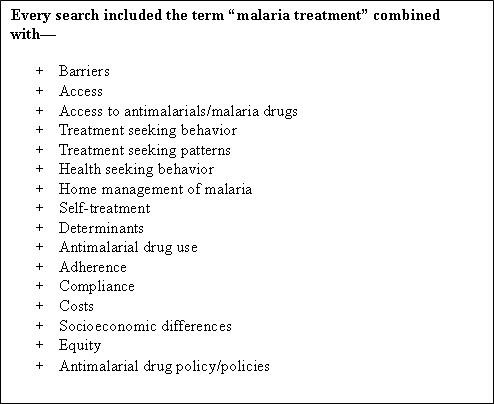
**Key words used for the review**.

PUBMED (MEDLINE) and HINARI were the main data bases used to retrieve papers included in this review. A summary of the search terms and strategies applied is provided in Figure 1. For unpublished data, individual researchers were contacted and reference lists of all studies retrieved, checked to identify any other relevant work. Websites of organizations working in malaria control, such as the DOMC, WHO, Management Sciences for Health and websites that aggregate information (for example, Eldis and ID21) were also included. Key variables of interest to the review included sources of treatment, types of medicines used to treat malaria, timing of treatment, costs of treatment, and determinants of access.

### Description of studies included in the review

A total of 39 articles were reviewed. The studies applied different methodologies and investigated different variables of interest making it difficult to arrive at a strict inclusion/exclusion criterion as would be expected in a systematic review. Consequently, the review included any study that focused on at least one variable related to any of the dimensions of access defined earlier. The majority of studies (n = 28) were carried out in rural districts; four were carried out in both rural and urban settings; and the remaining studies were desk reviews of malaria treatment policies. Sixteen studies focused on children as their population of interest. Most of the studies were quantitative; only three studies incorporated a qualitative component. The studies differed in methodology including cross-sectional surveys, longitudinal surveys, panel surveys, interventions, and desk reviews. They also differed in terms of when they were conducted: 10 were conducted before 1998 when the first line drug was chloroquine (n = 10); 20 we conducted between 1999 to 2005 when SP was the drug of choice, and only nine were conducted after 2006, the year that the government changed the treatment policy from SP to AL.

Studies explored various variables of interest ranging from physical access to clinical services, quality of pediatric case management, cost of malaria treatment, malaria management practices at hospitals, malaria diagnostic practices, and medicine dispensing practices. Most studies were not designed specifically to understand access, but they provided useful information on key variables of interest. The majority of studies (n = 25) were conducted at the community level.

Studies included in this review define malaria differently. While most studies used self-reported fever to indicate malaria, others tested for the presence of parasites, while others did not provide any information on how malaria was defined. For the purposes of this review all relevant studies were included, irrespective of how malaria was defined. The terms fever and malaria are used interchangeably.

## Results

### Sources of treatment

Several studies documented the pattern of treatment-seeking for malaria. The results indicated that treatment sources included both the formal and the informal sectors, with a few fevers left untreated. Drawing a line between the formal and informal sector is difficult due to the heterogeneity of services provided, and the nature of regulatory systems, that do not always provide a clear cut view in terms of what is formal or not [23]. For operational purposes in this review, the formal sector refers to the use of both private and public health care providers including dispensaries, health centres, clinics and hospitals. It involves consulting a health provider for illness diagnosis and treatment. The informal sector refers to the use of drug sellers (particularly shops) that do not require a licence to sell over-the-counter (OTC) drugs, although they may sell prescription drugs illegally. Drug sellers do not always provide professional consultation because they may not have appropriate training or qualifications. The informal sector also includes community health workers and traditional healers.

The proportions of fevers not receiving any type of treatment ranged from none [24] to 25 percent [25]. In general, treatment-seeking patterns were similar across settings and age groups, although children were more likely than adults to be treated by formal health providers, because malaria is perceived to be more severe in the former [26]. The main types of treatment included: self-treatment using drugs bought from private sector shops or left-over drugs from a previous illness episode, treatment received at private and public health facilities, traditional remedies taken at home, and use of traditional healers.

Table 2 summarizes the proportion of fevers treated through different sources of treatment, while Table 3 presents a summary of the sources of first actions for fevers. Most fevers were treated by adopting only one action. For example, in Gucha and Kisumu districts, 87 and 75 percent of all fevers, respectively, were treated with one action [27,28], while only nine percent of fevers in Kilifi district were treated using more than two actions [11]. A few studies reported the use of multiple treatments, with a typical pattern of self-treatment, followed by a visit to a health facility or traditional healer [24,26,28-31].

**Table 2:** Summary of treatment-seeking patterns as reported in different settings

**Reference**	**Formal**	**Informal**	**Traditional**
**Treatment-seeking patterns as % of all *treated *fevers**			

[7] (Children)	47	53	N/A*

[7] (Adults)	57	43	N/A

[37]	54	46	N/A

[45]	35	62	1

[27]	30	47	12

[34]	74	5	N/A

[44]	28	64	N/A

[29]	16	75	10

**Treatment-seeking patterns as % of all *reported *fevers**			

[37]	39	46	N/A

[36]	59	17	4

[28]	26	47	N/A

[26]	43	47	N/A

	38	30	32

[30]	30	32	25

[35]	55	32	7

[31]	25	59	7

[25]	14.3	83	3

[48]	44	55	N/A

*N/A = information not available.

**Table 3:** Percent of fevers *first treated *at various sources of treatment

**Reference**	**Informal**	**Public formal**	**Private formal**	**Formal (not categorized)**
	48	7	38	N/A

[7]	47	N/A	N/A	53

	57	N/A	N/A	43

[37]	33	30	9	N/A

[28]	47	N/A	N/A	26

[26]	77	N/A	N/A	N/A

[27]	67	N/A	N/A	38

[34]	12	74	15	33

[35]	38	32	24	N/A

[44]	65	N/A	N/A	N/A

[25]	86	9	6	25

[29]	85	17	N/A	N/A

Self-treatment using OTC drugs was the most common first action taken in response to malaria [18,24,26-32]. The results from different parts of the country show that the proportion of fevers first treated at home was high, but considerable variations existed between settings. In Kisii district, for example, the proportion of fevers first treated using drugs at home was 83 percent [24], while in Baringo district, only five percent of first actions involved self-treatment [33].

Use of the formal sector as a first source of treatment for malaria was less common, often comprising less than half of all actions [18,24,26,27,30,31,34]. A few studies have reported the formal sector as the main source of treatment, although the percentage of fevers that were first treated in the formal sector was low [35,36].

### Types of anti-malarial medicines used

The type of medicines normally taken to treat fevers was hardly documented in the studies reviewed. The few that classified the medicines taken reported that collecting these data was difficult due to the wide range of anti-malarial brands and other medicines in the market (for those self-treating), and because people do not always know the type of drugs they receive at health facilities [37]. Moreover, medicines in public health facilities are unlabeled and are normally dispensed from unlabeled containers, which makes it difficult for researchers to categorize the drugs during data collection [38].

Table 4 shows that the proportion of fevers treated using anti-malarials ranged from 23 percent [6] to 91 percent [39]. The proportion of fevers treated with government-recommended anti-malarials was much lower in all sectors, although people seeking treatment from the formal sector were more likely to receive the recommended anti-malarials than those in the informal sector. Overall, individuals who self-treat malaria use antipyretics more often [26,30,31,36,40-43]. A nationally representative survey reported that only 28 percent of children with fevers took anti-malarials. Of those children that received anti-malarials, 11 percent took SP, the nationally recommended drug at the time; 10 percent took amodiaquine, and three percent took quinine [40]. In Gucha district, only one-third of individuals reporting a fever received anti-malarials as part of home-based treatment, but only 27 percent of them took SP, the recommended treatment at the time [27]. In 2002/2003, the proportion of fevers receiving SP in public outpatient departments was estimated as 67 percent. Of these, 84 percent received the nationally recommended dose [39]. A recent longitudinal study found that amodiaquine was the most commonly used anti-malarial in both the formal and informal sectors, and although 31 percent of fevers were treated with anti-malarials at some point, only 10 percent were treated with AL, 95 percent of which was obtained from the formal sector [44].

**Table 4:** Use and source of anti-malarials for treatment of fever

**Reference**	**% of fevers treated with anti-malarials**	**% of fevers treated using first-line anti-malarial**	**Source of anti-malarials**
[7] (Children)	23	N/A	Informal

[7] (Adults)	37	N/A	Informal

[37]	31	N/A	Formal & informal

[41]	28	11	Formal & informal

[45]	31	10	Formal

[43]	33	27	Formal & informal

[26]*	47	3	Informal

[9]	48	90	Informal

[31]	29	N/A	Informal

[16]	N/A	1	Formal

[29]	58	12	Formal & informal

[11]	65	22	Informal

[40]	91	67	Formal

	77	55	Formal

N/A = information not available.

*Indicates the percentage of fevers given anti-malarials at home. Study does not include the percentage given anti-malarials at health facilities.

### Adherence to treatment

Assessment of treatment adherence was limited because very few studies provided data on use of medicines, and none were specifically designed to measure drug adherence. Nevertheless, the results indicated that inappropriate use of medicines was common among individuals using both the formal and the informal sector. In Western Kenya, for example, the appropriate use of drugs was only reported in 12 percent of patients treated at home, while in Kilifi and Malindi districts, only two percent of children given chloroquine bought from shops received an adequate dose [45]. Bungoma district also reported high levels of non-adherence, where despite 43 percent of ill children being treated at a health facility, 58 percent of caretakers stopped treatment after the children showed signs of recovery [25]. Elsewhere, 55 percent of mothers who received anti-malarials at health facilities did not follow instructions on how to use the medication, and none asked for clarification because they feared the health workers [30]. While none of the reviewed studies documented levels of adherence to AL in Kenya, the high level of non-adherence to previous anti-malarials suggests that adherence to ACT is also likely to be poor because of complex dosage patterns and the comparatively high cost. One of the few studies exploring adherence to ACT in sub-Saharan Africa reported 61 percent non-adherence among children receiving ACT in a health facility, and about 40 percent of the caretakers could not state the correct dosage for the medicine moments after they had received the drugs from the pharmacy [46]. Children whose parents had received some education and caretakers who spoke the same native language as the pharmacist were significantly more adherent.

### Timing and cost of malaria treatment to households

The timing of treatment is an important factor in managing malaria effectively. Table 5 provides a summary of the number of days taken before seeking treatment. Overall, the results reveal that delaying treatment was common; only a few cases were treated within the recommended first 24 hours; and that the number of days taken before seeking treatment differs by treatment source. A median delay of two days was reported, with only five percent of fevers treated with anti-malarials within the first 24 hours [36]. The number of days taken before seeking treatment in the formal sector was higher, with most people contacting the formal sector after 48 hours [11,25,26,30]. Individuals who self-treated were more likely to take medicines within the first 24 hours of symptom onset compared to those who sought treatment from the formal sector [18,25,28,31,43].

**Table 5:** Mean number of days taken before seeking treatment by source of treatment

**Study**	**Formal**	**Informal**	**General**
[37]	N/A	N/A	2 *

[43]	2	N/A	N/A

[27]	2	0-1	N/A

[31]	3	1	

[29]	N/A	N/A	1

[12]	4 (private clinics and government dispensary)	2	N/A
			
	9 (district hospital)		

[26]	3	N/A	N/A

*Median

Regarding costs, few studies estimated the costs of malaria treatment to households or to the health system. Comparing findings from the studies identified posed a challenge due to methodological differences in valuing costs and the variables explored. However, the results indicated that households spent a significant amount of money on malaria treatment. In Rusinga Island, the median expenditure for treating sick children with malaria was estimated as US$ 3.1 [47], while mean treatment costs were estimated as US$ 0.3 for the first action and US$ 0.1 for the second action in western Kenya [28]. Mean spending for an acute malaria episode amounted to US$ 3 [18]. Drug costs comprised the largest share of over 80 percent in all studies, while transport costs were relatively low.

A study that estimated the median cost of drugs for one treatment purchased in retail outlets reported a mean cost of US$ 0.2, while the median cost of drugs for one treatment from a private hospital was US$ 3 [36]. The average retail price of adult doses of SP and amodiaquine purchased from the Kenyan retail sector was estimated as US$ 0.4 and US$ 1 respectively [48], while the median cost of AL was US$ 11. Direct cash expenditure per fever was estimated at $ 0.4, and a total cost of US$ 2 (including direct costs) for uncomplicated fevers treated according to the national guidelines i.e. prompt treatment with AL. It is worth noting that the average costs to households of treating malaria are likely to increase substantially if AL is availed in the informal retail sector.

### Factors influencing access to prompt effective treatment

Although a number of studies reported on factors influencing access to malaria treatment, access was not their primary focus. Of the studies reviewed, only one study was specifically designed to understand barriers to access. A variety of interrelated factors affect prompt and effective treatment of malaria. They include demand, supply and policy-level determinants.

#### Demand-side determinants

Demand-side determinants refer to factors at the individual and household level that influence the ability to initiate and successfully complete appropriate malaria treatment. Some demand-side factors that hinder access to effective treatment include: the community's poor perceptions of quality of care, their lack of trust in health providers, and their perception of the effectiveness of drugs provided in government health facilities [28,49].

Perceptions of causes of illness and differences in biomedical and traditional illness concepts can hinder prompt access to effective treatment. For example, convulsions may be a sign of severe malaria, but in some parts of Kenya, they are associated with supernatural powers, which require the use of traditional healers and therefore not amenable to western pharmaceuticals [26,30]. While people's understanding of malaria has improved over the years, and the use of traditional healers for malaria treatment has reportedly declined [11], such beliefs may limit the acceptance of treatment regimes. Continuous education and awareness creation of the causes and treatment of malaria should be maintained.

Perceptions of illness severity and duration of illness may prolong the period between treatment and onset of symptoms. The longer the period of illness, the more likely that the caretaker or patient will seek treatment [24,26,30,43,50]. People tend to adopt a wait-and-see attitude when symptoms are perceived as less severe, only seeking treatment when symptoms persist. Fever among children prompts quicker action, which explains why a large proportion of fevers were treated either at home or at a health facility [18,24,26,51]. Care-seeking was also shown to differ between seasons due to changes in cash availability and perceptions of illness severity between wet and dry seasons [11,18]. Cultural factors, too, have been shown to influence timing of treatment. Women have limited power to make decisions, while men control resources, making it difficult for mothers to seek prompt treatment for their ill children [26,43].

Communities' perceptions of different types of treatment may hinder prompt access to the most effective treatment. In Bungoma district, the community believed that SP was too strong for children under one year and therefore opted to buy alternative, but ineffective medicines, to protect children from the perceived strong effects of the drug [52]. In Kilifi district, uptake of SP was constrained by widely held beliefs within the community that chloroquine was still effective [8]. Elsewhere, health workers reported that they continued to prescribe SP and amodiaquine because patients demanded familiar drugs [53].

Effective treatment can also be influenced by simplicity of drug regimen, which is associated with higher levels of treatment adherence. In Kilifi district, only about 10 percent of people who purchased amodiaquine from shops took the drug appropriately [8]; but the introduction of SP resulted in higher rates of appropriate medicine use, a factor partly attributed to SP's simpler single-dose regimen.

Ability to meet the costs associated with a particular treatment is an important factor influencing access and choice of treatment. Even where malaria treatment is supposedly free (in government health facilities), individuals still incur costs, such as transport and laboratory services [17]. People often start with a cheaper treatment, then choose a more expensive option as the illness persists or when funds become available [24,26]. In agricultural communities, the seasonality of cash income may prevent households from seeking treatment on time [18].

#### Supply-side determinants

Supply-side determinants refer to health system factors that deter people from obtaining appropriate treatment. The degree to which services are available and acceptable to the population plays a critical role in ensuring prompt access to effective treatment.

Distance from health facilities is a critical factor influencing the use of the formal health care sector [18,27,33,54]. In Baringo district, 74 percent of households reported that public health facilities were close to where they lived, which was the main reason why they chose the formal sector as the first source of malaria treatment [33]. In many areas in Kenya, however, the distribution of public health care facilities is poor. Noor *et al *noted a reduction in the number of patients using formal health services as their distance from health facilities increased [54], while one study reported that one-third of patients who self-treated said that they would have sought treatment from a health facility if it were near [29]. Not surprisingly, urban residents have better access to formal health care services than their rural counterparts [26,55].

Another supply factor hindering access is availability of medicines. Often, public health facilities do not have the recommended anti-malarials in stock. Lack of medicines in the formal sector contributes to people buying drugs OTC, where the quality of drugs is less controlled and information on dosage is not often provided. Health workers and community members reported that public health facilities suffered from chronic drug shortages due to delays in drugs deliveries from the central level and the failure to adjust drug quantities to suit seasonal fluctuations in disease burden [17]. However, health workers did not always prescribe the appropriate anti-malarials to patients, even when the drugs were in stock [17,37,39]

Failure to prescribe the recommended medicines has been shown in a recent study exploring prescribing practices following the change of treatment policy from SP to AL. A low level of prescriptions of the nationally recommended drug was reported. Only 26 percent of children needing treatment with AL according to national guidelines received a prescription for this drug; 39 percent received amodiaquine; four percent received SP; eight percent received other anti-malarials; and 23 percent left the facility without any anti-malarials prescribed, although their symptoms indicated the need for anti-malarials [37]. In cases where AL was prescribed, dosages were more likely to be correct compared to the more common drugs of amodiaquine and SP [39,56]. The authors highlighted the need for more case-management interventions--including training and supervision--to ensure that all febrile children receive the treatment as recommended by the national guidelines. However, they noted that the short time between policy implementation and the study did not allow for any conclusions regarding supply of AL in government facilities.

A follow-up study exploring why health workers did not prescribe AL, despite the drug being in stock at the health facilities, identified various reasons for non-adherence to the treatment guidelines, most of which were related to health workers responding to general health system weaknesses [53]: (1) insufficient supply of AL raised fears of stock-outs if health workers prescribed it to all deserving cases; (2) although AL was provided for free, it was considered expensive resulting to health workers assessing which cases deserved to receive the drug; (3) continuous supply of amodiaquine to health facilities after the policy change, which led to health workers' confusion about prescribing it; (4) lack of follow-up supervision after training; (5) staff shortages and a high workload; (6) patients' preferences for SP over AL because of its simple dosage and; (7) cumbersome guidelines and contradictory training messages that confused health workers.

Other supply factors are around quality of medicines. A large number of unregistered and substandard pharmaceuticals circulate in the Kenyan market. An audit of 880 retailers (drug shops and pharmacies) identified 215 anti-malarial brands in circulation in 2002 [48]. Of these, only half of the SP and amodiaquine drugs were registered with the Pharmacy and Poisons Board (PPB). Unregistered drugs pose a danger to treatment because their safety, efficacy, and quality cannot be guaranteed. Amodiaquine was the most widely available anti-malarial in the retail sector, despite being a prescription-only medication, while SP, the first-line drug at the time of the survey, was only available in 29 percent of the outlets. The authors argued that although a lack of regulatory enforcement in Kenya's drug sector may be responsible for the pattern observed in the retail market, limited community awareness of the treatment policy may have contributed to the market for non-recommended drugs. In addition, a wide range of products available to the community can create brand confusion, which may lead to unintentional repeated doses of the same drugs.

#### Policy-level determinants

Policy-level determinants can result at the national and local levels, related to changing the first-line drug, staffing, or financing public health facilities. Policy complexities and unclear messages can undermine the potential of a well-intended policy to promote quality case management. A change in drug policy faces many challenges from both the supply and demand perspective [8].

The challenges of changing drug policy in Kenya are well documented. Adequate preparations, ensuring that pharmaceutical industries are engaged in the discussion regarding policy change during the initial stages to enable them plan and adjust their manufacturing strategies in line with the new policy to assure product availability, are some of the factors that can promote smooth policy transition [57]. A policy aimed at exempting malaria patients from paying for treatment at public health facilities in Kenya has had limited impact [17]. While this policy aims to reduce barriers to access for malaria patients by reducing costs, health workers expressed concerns about the practicalities of implementing it. In settings where malaria is the main cause of morbidity, facility managers reported that exempting malaria patients from paying facility registration fees would lead to loss in revenue. Some facilities opened privately operated laboratories, and health workers have been reported as reluctant to prescribe AL before the patient takes a parasite test. While laboratory services are essential for identifying malaria, particularly among older children and adults, having privately owned laboratories in health centers and dispensaries increases costs to households and thus barriers to access.

As mentioned previously, home treatment increases the proportion of ill individuals receiving anti-malarials promptly, but the change in treatment policy to ACT may affect the effectiveness of the anti-malarials that people buy and take at home. ACT is not currently available in the informal sector, which was an important source of anti-malarials before the policy change, and amodiaquine remains on the shelves as an alternative to those seeking treatment from the informal sector. In an effort to stem the circulation of ineffective anti-malarial monotherapies in the market, the PPB issued a circular to local manufacturers and importers of these products to stop the manufacturer and importation of ineffective anti-malarial monotherapies by end of March 2008. The extent to which this was implemented is not clear.

## Discussion

Self-treatment using OTC drugs will remain a norm in household treatment for malaria. All studies in this review, except for one, identified use of OTC drugs as the most common first action. Self-treatment for malaria has been reported in many parts of sub-Saharan Africa [58-62], and various interventions have been put in place to promote appropriate use of anti-malarials purchased through the informal sector [7-9]. Shopkeepers training interventions have been shown to be effective in terms of costs and in reducing inappropriate use of anti-malarials [6-9]. Nevertheless, controlling the quality of anti-malarials sold in the retail sector is difficult due to the heterogeneity of the shops selling OTC drugs, clients demands and the profit maximization goal that defines the existence of the private sector [63]. Currently, the informal sector (shops and community health workers) are not allowed to sell AL, although the same are available in the formal retail sector (registered pharmacies). To improve access to effective anti-malarials, AL should be made available through the informal retail sector. Currently, the DOMC is considering introducing AL for the home management of malaria. Such an approach should be accompanied by appropriate training programmes, an educative social marketing campaign to promote awareness of the new drugs, and should be well designed to meet communities' needs.

Often the drugs bought to treat malaria through OTC are inappropriate and antipyretics remain the most widely purchased drugs to treat malaria. Even with the treatment policy change from SP to AL, the review identified amodiaquine as the main type of anti-malarial bought from the informal sector, in addition to being prescribed widely by health workers [44,48,64]. Some anti-malarials available in the market are substandard or counterfeit [9,48,65], which is particularly worrying, because substandard drugs not only endanger lives, but they also jeopardize future malaria treatment strategies. Poor enforcement of pharmaceutical sector regulations can be a major threat to anti-malarial effectiveness and provide a gateway to drug resistance. Controlling the quality of anti-malarials provided through the private sector remains a challenge for Kenya's drug regulatory agency. Nevertheless, it is important to ensure that anti-malarials available in the market are not only registered and meet the required standards, but that monitoring is done to ensure safety, quality and efficacy post-registration.

When people access the appropriate anti-malarials, poor adherence to dosage is common. Although the review did not identify any study that investigated adherence to AL in Kenya, there are concerns that levels of non-adherence are likely to be high and that this will not only result to treatment failure, but will also encourage the development of drug resistance [66].

Widespread appropriate use of anti-malarials will occur only if significant efforts are directed towards educating the community. Key messages that should be incorporated in education campaigns should include the benefits of using the first-line recommended anti-malarials correctly, the dangers of incorrect dosage, and the types of anti-malarials that are licensed by the PPB. Current strategies, such as use of pre-packaged AL for weight, may help increase adherence and improve communication between health workers and patients. Since cost is a major barrier, the government together with donors should consider subsidizing AL provided through specific accredited retail drug shops operating in malaria-endemic settings. This, however, requires substantial resources and commitment from the government and donors to ensure sustainability. Initiatives to subsidize ACT are already in place (for example, the Affordable Medicines Facility for malaria), but the sustainability of their support to disease endemic countries, including Kenya, is uncertain. Other potential interventions include improving communication between health workers and patients, for example through using pictorial aids, when dispensing anti-malarials to ensure that patients understand the correct dosage [67]. Although the review did not focus on access to prevention, it is evident that campaigns to promote the use of ITNs have achieved amazing results [4]. While prevention plays an important role in reducing malaria morbidity and mortality, interventions that address treatment are equally important. In Kenya, information on ITNs use is easily available through the media and one is likely to find posters at health facilities and other areas advocating for the use of ITNs. Similar efforts towards appropriate use of anti-malarials are needed.

The review has shown that health workers do not always prescribe the appropriate anti-malarials or doses to patients [37,64,68]. Previous research on health worker prescribing interventions has yielded mixed findings, with some showing significant improvements in case-management and others showing no effect at all. Regardless, health workers require support to improve their prescription practices, particularly when policy changes.

Health system factors play an important role in promoting or hindering access to effective treatment. These interrelated factors (for example, drug availability, provider practices, quality of care and non-functional policies) should be addressed as part of the system as a whole. While the DOMC may address certain barriers of access, it might not be in a position to influence providers' behaviour or address the deeper rooted problems associated with high workload and poor remuneration. Improving access to effective case management therefore, requires that the Ministry of Health works together with the private sector, the civil society and development partners towards achieving a partnership that ensures the services provided in both the public and private sector are in line with the national guidelines.

### Priorities for further research

To inform future policies and interventions various research areas should be given priority. Information on drug use patterns following policy change from SP to AL is lacking. Only one study explored use of anti-malarials following the implementation of the new policy, and while this study provided useful insights into the range of medicines used to treat malaria, it was conducted within the first year of policy change (2006/2007). Considering the supply of AL to the public sector was only initiated in September 2006, additional data on the effect of the policy change over time are required. A survey exploring the nature of the market for anti-malarials would complement household- level data on drug-use patterns.

Adherence to treatment is a topic that has not been explored extensively. Adherence to ACT may pose a challenge, especially for the retail sector where business and cost-saving strategies are key for the retailers and households respectively. Research to evaluate adherence to AL would be useful for informing interventions aimed at promoting effective use of anti-malarials.

Provider prescribing practices require further investigation. Further exploration of factors influencing providers' behaviour and their perceptions regarding new treatment guidelines would help inform the design of interventions to improve diagnosis and prescribing practices.

Finally, more qualitative studies are needed to explore and understand barriers to access. Unless the whys' and hows' behind treatment-seeking patterns, irrational drug use, and provider behaviour are well understood, effective case management and achieving prompt access will remain a challenge. Such studies should be designed to explore all dimensions of access and how they interrelate at the demand, supply, and policy levels.

### Limitations of the review

There are several limitations of this review. First, attempts to compare the evidence across studies were complicated by differences in methodologies, populations of interest, and sampling methods. Second, the majority of studies were conducted in rural settings and while rural populations might face more barriers to access than their urban counterparts, similar data on urban settings were lacking. Third, access to malaria treatment was not a primary aim for the majority of studies, and was thus not fully explored. Finally, most studies were conducted at a time when the recommended first-line anti-malarials in Kenya were SP or chloroquine. Drug use patterns may be different with the use of AL; information on drug use reported in this review should be interpreted with caution.

## Conclusion

Achieving the Abuja target on access to effective anti-malarials within 24 to 48 hours remains a challenge for Kenya. Additional efforts are needed to ensure that people have access to the effective anti-malarials, and that high levels of adherence to treatment are achieved. The government, with support from donors, should invest adequately in mechanisms that promote access to effective treatment. Such approaches should focus on factors influencing all dimensions of access and will require the cooperation of all stakeholders working in malaria control.

## Competing interests

The authors declare that they have no competing interests.

## Authors' contributions

JC was involved in the conceptual design of the review, collected and summarized all the articles and documents for this review, drafted the report from which this paper is based, and was responsible for the overall writing of the manuscript. TA drafted the initial manuscript from the report. TA and JN assisted with retrieving and summarising findings from the papers. DM, EJ, WA, AN, RM provided information on policy discussions around the DOMC's strategy on improving access to anti-malarial medicines and the implications of the study for national roll-out of the ACT policy and substantially revised the manuscript. GT was involved in writing the initial report and of the manuscript. AA conceptualized the study and was involved from the overall design and writing the report on which this manuscript is based. He contributed significantly to the writing of the manuscript and provided the policy perspective for the review. All authors read and approved the final manuscript.
